# Quantifying mating success of territorial males and sneakers in a bower-building cichlid fish

**DOI:** 10.1038/srep41128

**Published:** 2017-01-27

**Authors:** I. S. Magalhaes, A. M. Smith, D. A. Joyce

**Affiliations:** 1 School of Environmental Sciences, University of Hull, Cottingham Road, Hull, HU6 7RX, UK; 2 University of Nottingham, School of Life Sciences, University Park, Nottingham, NG7 2RD, UK

## Abstract

The strategies and traits males evolve to mate with females are incredible in their diversity. Theory on the evolution of secondary sexual characters suggests that evolving any costly trait or strategy will pay off and stabilise in the population if it is advantageous compared to the alternative less costly strategy, but quantifying the relative success of the two can be difficult. In Lake Malawi, Africa, there are >200 species of cichlid fish in which the males form leks and spend several weeks per year building sand-castle “bowers” several times their size. We tested the idea that a less costly “sneaking” strategy could be successful by quantifying the mating success of bower-holding versus non-bower-holding males. We PIT-tagged every fish in a semi-natural experimental set-up and placed tag-readers on the side of bowers to determine which fish held a bower. We then genotyped the eggs removed from females’ mouths to assign paternity of each egg. Broods were fathered by up to 3 different males. Although paternity was mostly assigned to males that held a bower, a small number of males who did not own a bower were more successful than some of those that did, indicating a role for an alternative strategy in these bower builders.

The investment by males in ornaments and armaments is one reason that organismal phenotypes are so diverse. Female preference for certain characteristics means that sexual selection drives males to develop traits specifically to attract mates, i.e. secondary sexual characters; on which females base their mate choice^1^. Males that provide no parental care can devote their reproductive efforts to mating resulting in intense competition over mates^2^. This is particularly visible in species that form leks, where males aggregate and engage in competitive displays to attract females. Male mating success in leks is typically skewed towards a small number of individuals, with high social dominance^3^, or a more central position^4^, yet the skew varies according to lek size.

One outcome of strong competition for mates is the development of alternative reproductive tactics (ARTs)^5^,^6^,^7^. An example of this is “sneaking” behaviour, whereby a male will attempt to mate with a female that has chosen another, usually more dominant male, as a partner. Lekking species, as any species with a highly reproductive skew towards a few dominant individuals, might be predicted to exhibit ARTs more often, since this would be advantageous to males with little chance to reproduce otherwise. In some lekking species males build structures that act as non-morphological extensions of their phenotype (i.e. extended phenotypes^8^). Such structures act as mate choice signals and this in particular may facilitate ARTs because of the substantial cost added by the construction and maintenance of such a structure, which might be prone to take-over by a conspecific male^9^. Theoretical work on the evolution of secondary sexual characters suggest that evolving a costly strategy, such as having a territory that an individual needs to defend and that will more likely expose him to predators, will pay off and stabilise in the population only if it gives an advantage compared to the alternative less costly strategy^2^. The prevalence of ART in lekking species, particularly those using extended phenotypes is however understudied^10^.

Here we used a species of bower-building haplochromine cichlid (*Nyassachromis* cf. *microcephalus*) from Lake Malawi to quantify the prevalence and relative success of ARTs in a lekking fish species, and test the hypothesis that males who invest in building and defending a bower have higher reproductive success than those that don’t. Approximately 200 species of bower-building haplochromine cichlid have evolved in the last ~5 million years in Lake Malawi^11^,^12^. These species congregate in leks, and build and defend sand-castle structures, termed “bowers” because of their apparent lack of utility beyond male signalling, analogous to the structures of bower birds. They are mouth-brooding cichlids; females spawn and then pick up the eggs in their mouth, while males simultaneously release sperm over the eggs. The fertilized eggs remain in the females’ mouths for about three weeks until they hatch and the fry are released into the water. Mouth-brooding females gain no direct benefits, such as food, protection, or territorial space from their mates. Bowers tend to be species specific, and are used in both direct female choice^13^,^14^,^15^, and male-male signalling^16^,^17^,^18^.

Studies on species of sand-dwelling cichlids of the genera *Copadichromis* and *Otopharynx*^19^ have indicated females mate with up to three males in a single spawning^20^, and in wild *Copadichromis cyclicos* broods, between 1–6 males were found to have fathered a single brood^21^. However, multiple mating is not necessarily indicative of ARTs, particularly in species in which females visit leks and may choose a number of mates. Therefore quantifying the paternity that is contributed by non-territorial males is necessary in order to assess whether sneaking occurs in these fish. Sneaking behaviour has only rarely been observed in bower-building species^22^,^23^ so the relative success of this tactic compared with bower building and defence is unknown. Given the apparent reproductive skew observed for this species^24^, we might expect ARTs to confer an advantage to males who are unable to build and defend territories.

We conducted a large semi-natural experiment in which we tracked fish movement and bower ownership, and employed microsatellite markers for paternity assignment of broods to (i) determine whether sneaking behaviour is prevalent, and (ii) quantify mating success of territorial bower holders when compared to males that do not hold a bower.

## Results

### Bower ownership

We conducted 5 trials, each lasting between 19 and 26 days (see Table S1 for detailed schedule of each trial and video S1 for an overview of the fish in the experimental pool). Computational problems with the system meant we could not keep the tag readers on for 24 hours a day, but we recorded data for 696 hours across all trials and readers. Over the five trials we monitored a total of 21 bowers (3 to 5 per trial) and obtained 138,547 reads. Bower ownership was assigned on a daily basis using PIT tag reading antenna placed over one of the sides of any bower and counting the number of reads per tag per day. Each bower was assigned to the male with the most reads per day, since bower-holding males typically spend their time swimming just above their bower, maintaining and defending it. We were able to detect that out of 21 bowers, 15 had their ownership consistently assigned to a single male for the whole time the tag-readers were on, 5 bowers were assigned to 2 males and 1 bower was owned by 3 males on separate days in one trial (Table S2). Two males appeared to dominate more than one bower within a trial: in trial two male 24-987B had the most read male tag on 3 different bowers on 4 separate days and in trial 4 male 24-8506 had the most read tag on two different bowers on two separate days. Twenty four males were used in more than one trial and only two of these were dominant in all trials they were part of.

### Paternity assignment and relative male mating success

In order to quantify male mating success we genotyped 20% of each brood of eggs (but not less than five eggs if the brood size was larger than five) removed from females’ mouths at the end of each trial. A total of 239 eggs from 35 broods were assigned to 22 fathers across all trials, with an average of 4.4 males fathering offspring per trial (see Table 1, and Tables S4 and S5 for detailed results of the paternity analyses and assignment levels). Based on 20% of offspring being genotyped, females mated with an average of 1.51 males, and a maximum of 3 males (of 15 possible in each trial). Of the 35 broods, 20 had all eggs assigned to a single father, in 12 broods eggs were assigned to two different fathers and in 3 broods to three fathers.

**Figure 1:**
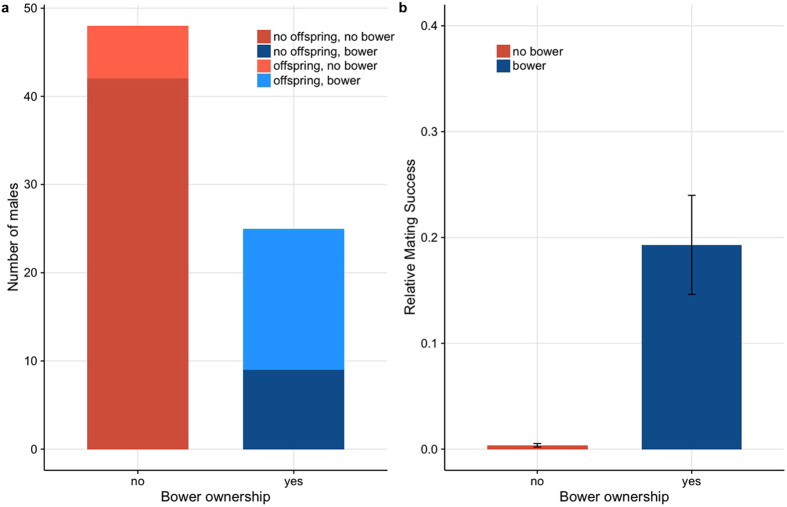
(**a**) Number of males with and without bowers (red and blue respectively) who fathered offspring (lighter coloured section) and who didn’t (darker coloured section) and (**b**) Mean individual relative mating success of bower holding males (blue bar) and non- bower holding males (red bar). Black bars represent standard errors (unpaired t-test, n1 = 25, n2 = 48, t = 4.044, p (one tailed) < 0.001).

Bower-holding males had significantly higher mating success than males that had no bower assigned to them (unpaired t-test, n1 = 25, n2 = 48, t = 4.044, p (two tailed) < 0.001) (Fig. 1, Table S6). Out of 25 males that held a bower at some point in the experiment, 16 (64%) were assigned paternity of a whole or part of a brood, and on average 96% of offspring were fathered by bower holders. Of the 48 males that did not hold a bower, 6 (12.5%) had offspring assigned to them. All the offspring sired by these 6 males were part of clutches with mixed paternity, and the proportion of offspring they sired in each clutch ranged from 0.07 (1 out of 14 eggs) to 0.19 (3 out of 16 eggs). The fact that the tag-readers were not on for the whole length of each trial meant that some males could have had a lower number of reads because they were holding a bower while the tag-readers were not on. In order to account for this we repeated the t-test including the second most read male as bower holders (Table S2), but the result was identical (unpaired t-test, n1 = 31, n2 = 42, t = 3.829, p (two tailed) < 0.001, Figure S2). Out of the 6 fish that were moved from not holding to holding a bower only one had fathered offspring.

We also assessed the effect of bower-holding on mating success, relative to the effects of other male traits and found no effect of male weight, size or condition factor on male mating, but found a significant effect of holding a bower (Table 2).

Males used in more than one trial had a significantly higher mating success when they held a bower than when they didn’t (paired t-test, n = 10, t = −2.88, p (two tailed) = 0.018).

## Discussion

We used a semi-natural experiment to recreate a *Nyassachromis* cf. *microcephalus* lek in order to monitor bower ownership, quantify paternity, and assess the role of ARTs in lekking, bower-building cichlids. The building of bowers that act as intra and intersexual signals characterizes a whole radiation of sand dwelling cichlid fish from Lake Malawi^12^,^25^, with bower shape being used to define several closely related species^26^. Wild males invest a considerable amount of time and effort building their bowers, depositing a mouthful of sand on the bower approximately every 15 seconds during the courtship period^14^. Why these relatively small fish spend so much time and energy building bowers with base diameters more than eight times their own standard length has been in question for decades^13^,^16^,^22^. Most studies, however, have focused solely on the males that have bowers and the role bower size and location play on male mating success^13^,^14^,^17^,^22^. Even though solitary males are often seen passing by bowers and engaging in fights with bower-holding males or interrupting the mating process^16^,^17^,^18^, no study until now had addressed the advantage of holding a bower versus not holding one and quantified mating success of the two mating strategies. Our genetic assessment of parentage suggests that although bower holding males father the vast majority of the offspring, bower ownership was not a guarantee of mating success, reflecting the reproductive skew observed in the wild^24^. Such a skew may sometimes allow sneaking to be a more successful strategy than owning a bower.

An experiment such as this would be very difficult or impossible to carry out in the wild, but there are necessarily some limitations to our findings, which use a small number of individuals in a restricted environment where individuals were not monitored 24 hours a day. This last problem is particularly relevant for the interpretation of our results on the success of sneaking males. As the tag-readers were only working part of the time it is almost impossible to be certain that a male sneaker that fathered offspring was not actually holding a bower when he mated. This could lead to an overestimation of the number of sneakers that were assigned paternity. Nonetheless we find this possibility unlikely for almost all of the successful sneakers. Our tag-reader data suggest that males tend to maintain their bowers for long periods of time, not just for a few minutes or hours. Therefore, even if the tag-readers were not able to record the whole period of time a male held a bower for, it is likely that there would be a relatively high number of reads for that male. Of the 6 sneakers that were assigned paternity, only one had such a number of reads (the second highest number of reads for one bower). All others had very few reads and were not recorded over a bower for more than a few seconds in a row. It is therefore possible that one of the males we considered a sneaker might have held a bower, but we find it unlikely for the five other males.

Since it is very difficult to count non-bower holding males in the wild, their prevalence is not known and so the ratio of bower holding: non-bower holding males we created could be either too high or too low. This may lead to comparatively more or fewer sneaking opportunities than would be expected in the wild. Nevertheless, sneaking seems to be a viable strategy. In wild populations, bowers tend to be built in densely populated leks and central bowers are thought to have an advantage due to more female encounters^17^. Males with bowers in the periphery of the lek have more fights with heterospecific fish^18^ and may be more exposed to predators. It is possible that in the wild it may be more advantageous to be a sneaker than to build and maintain a bower in a location where attacks are frequent and female encounters low. This would explain the maintenance of a sneaking strategy in wild populations, despite a relatively low paternity likelihood.

Bowers are used by males to compete for the optimal positions on large leks preferred by females^16^,^24^ and are likely to be an honest indicator of competitive ability. Bowers used in indirect female choice^27^ may be a signal that has been co-opted for use in direct female choice, similar to bowerbird skrraa calls used in aggression and courtship^28^. Several bower traits, such as height are known to be used by females choosing among males who hold a bower^13^,^14^,^29^. Choosing males with bowers might be the first step of a sequential assessment of several bower and male traits used by females to evaluate the quality of their potential mates^15^,^30^. There are indications that bower construction in cichlids is an honest indicator of male quality^31^,^32^. When females can accurately assess male quality according to some signal, high quality males will be chosen, and there will be a greater incentive for lower quality males to switch to an alternative tactic^33^. Additionally, assessing male quality does not preclude the choice of sneaking males. However it is possible that a sneaker’s reproductive success derives from interrupting a courting territorial male and attempting to fertilize the female rather than being chosen by the female during courtship. Multiple paternity (with territorial or non-territorial males) may however allow a bet-hedging strategy by females, assuming there is some heritability in reproductive tactic^34^. The fact that males did not appear to consistently use one tactic or another between trials in the experiment indicates some plasticity, and the heritability of bower ownership or sneaking needs to be properly estimated before ARTs can be considered an evolutionary strategy upon which selection can act.

An unexpected result of our experiment was that, even though the majority of the bowers were assigned to one owner during a trial, some bowers changed ownership. Observational studies of bower-building cichlid species from Lake Tanganyika found that males that were removed from or deserted their bowers were quickly replaced by other males on the bowers^30^,^35^, with desertion of a bower being attributed to exhaustion resulting from bower maintenance and reproduction^30^. For Lake Malawi bower-building cichlids it is known that wild males temporarily abandon their bowers, usually during the afternoons, to feed nearby^14^,^22^, but replacement of one male by another as a bower owner had never been reported and past field experiments on this species have been carried out under the assumption that a bower was owned by the same male throughout the whole season. Our results indicate that, either due to desertion or competition, some bowers changed ownership during a trial. This finding, together with the fact that the same male did not always hold a bower across trials also suggest that building and/or holding a bower has an element of plasticity. Size and weight did not seem to reflect bower ownership, and male quality was something other than “condition” as we measured it. Fixed genetic differences in ARTs have been reported in several organisms^36^,^37^ (reviewed by ref. 38), and some cichlid species have ARTs that are fixed for life, whereas other studies have found that ARTs change throughout the life of a male with male size being important when acquiring and maintaining a territory and bigger males having the advantage^39^,^40^.

To conclude, our results show a large skew of paternity towards males who hold a bower, indicating that the costs of building and maintaining it pay off when compared to the alternative strategy of sneaking. However, paternity is most likely not constrained to bower holders, so a potential role for sneaky mating exists, as might be expected in a lekking system with high reproductive skew towards a few individuals. The use of an extended phenotype in sexual signaling may facilitate ARTs, and the independence of the signal from the body could select for plasticity in tactics because of the possibility of bower acquisition over a mating season.

## Methods

### Individuals used in experiments

Males and females used in this experiment were *Nyassachromis* cf. *microcephalus* laboratory bred F1s. They derived from a population of fish collected at Thumbi West Island in the Lake Malawi National Park (14°01′22 S, 34°49′24E) in 2007, and maintained at the University of Hull, UK. Females of this species are grey but breeding males are colourful with metallic blue heads and flanks, dark vertical stripes and yellow/blue on the mid body and black fins. The wild caught individuals occupied a shallow water lek at the shore of the Island.

Males and females were kept separately in stock tanks before the start of the experiment and were returned to stock tanks between trials. Each of these tanks was 180 × 40 × 45 cm, holding approximately 285 litres of water, and part of a 5,500 litre recirculation system through which 720 litres passes per hour, and that receives a 15% daily water change. Water temperature was maintained between 24–26 °C. Aquaria were illuminated on a 12D: 12 L regime using full-spectrum fluorescent tubes including UV light. Fish were fed once a day with dry food.

We used a total of 36 males and 84 experimental females over the 5 trials. Out of the 36 males, 12 were used only in one trial, 11 were used in two trials and 13 in three trials. Out of the 84 females, 58 were used in one trial, 15 in two trials, 10 in three trials and 1 in four trials. Fish used in the experiment were individually PIT (Passive Integrated Transponder) tagged, fin clipped and weighed by A.M.S. (For all trials, only mature, colourful and potentially reproductively active males were used. For males in the last three trials we also took their length (standard length, SL) and estimated their condition factor. Condition factor for each male was calculated as 100*weight (g)/length (cm)^2.76^ 26. In some cichlid species size and weight are characteristics used by females in male choice^41^ and this could confound the effect of holding a bower, so we used these measurements to control the effects of weight, size and condition factor on male success. The experiment was carried out in accordance with relevant guidelines and regulations established by the UK Home Office and was part of a UK Home Office licensed project (60/3295).

### Experimental setup and design

The experiment was conducted in 5 trials between July 2009 and April 2010. It was carried out in one circular above ground pool with 3.6 m diameter and 1.2 m height in a greenhouse at the University of Hull’s Botanical Gardens (video S1). There was a 20 cm depth of fine sand (3 tonnes) and the pool was equipped with a pump, biofilter, UV steriliser and heater. Water temperature was maintained between 23 and 25 °C. Tagged fish were allowed to swim freely and there was full interaction between males and females. Food was distributed three times per week. On the first day of each trial 15 males were introduced into the pool with 8–12 non-experimental females to stimulate males to establish territories and start building bowers. After 7–10 days, fish were removed from the pool, males immediately returned and 25 experimental females added. During the last week of each trial, a circular PIT tag reading antenna was placed over any bower that had been built (see Table S1 for detailed schedule of each trial and video S1 for an overview of the fish in the pool). These tag readers detect and record any PIT tags within 40 cm, approximately every second.

On the last day of each trial (19–26 days after the start) all fish were removed, and brooding females were weighed, standard length measured, and their eggs were removed from their mouth and preserved in 95% ethanol.

### Bower ownership assignment

We assigned bower ownership by assuming that the male with the most reads on a particular bower owned that bower, since males typically spend their time swimming just above their bower, maintaining and defending. We collected data for the longest periods possible, but computational problems with the system meant we could not keep the tag readers on for 24 hours a day (see Table S2 for details). Bower ownership was assigned on a daily basis by dividing the number of reads for each male by the total number of reads, and by the total number of male reads.

### DNA extraction and genotyping methods

We obtained a total of 45 broods from all the trials. However, as the tag readers were only working for part of the time the fish were in the tank, we discarded 10 broods that we thought might have been fathered before bower ownership was assigned. *Nyassachromis* eggs hatch after 5 days and the egg yolk is completely absorbed after 21 days. Extensive breeding and rearing of known broods means we can relatively accurately estimate the age of the embryo by the size of the fish compared to the yolk.

For each trial the mother, all 15 potential fathers and 20% of each clutch (but not less than five eggs if the brood size was larger than five) were genotyped at four microsatellite loci using fluorescently labelled primers: Ppun5; Ppun7; Ppun21^27^ and TmoM5^28^. Clutch size ranged from 1 to 75 eggs. The number of eggs genotyped varied from 1 to 19 eggs per clutch. Only three broods had less than five eggs genotyped. With a minimum of five eggs per clutch, the probability that an allele at a given locus was missed is 0.5^5^ per parent^29^. DNA was extracted using the HotSHOT method^30^ and microsatellite loci were amplified using the QIAGEN multiplex PCR kit according to the manufacturer’s instructions. Fragments were separated using a Beckman Coulter CEQ 8000 capillary sequencer and sized using the CEQ 8000 Series Genetic Analysis System (see Table S3 for summary statistics of the microsatellite data).

### Paternity assignment

As broods were removed from the mother’s mouth, maternity was unambiguous. Nonetheless, we checked for possible scoring errors by checking for mismatches in microsatellite alleles between mother and eggs. Paternity was assigned using Cervus^31^, which allows for inclusion of an input file with the identity of the mother. Paternity was checked by two complementary methods: (1) exclusion from paternity of males that had any mismatches in microsatellite alleles with an egg and (2) relaxed (80%) and strict (95%) confidence level. Possible scoring errors were considered by allowing a 0.01 error rate per locus.

### Relative male mating success

The mating success of each individual male relative to all other males in each trial (“Mating Success”) was calculated from the results of the paternity assignment using the equation 1^29^.


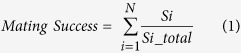


where *Si* is the number of offspring sired by an individual male per trial (i); *Si*_total is the total number of offspring sired by all males per trial (i). Mating success potentially ranges from 0 (a male did not sire offspring) to 1 (if a male was the only one that sired offspring in each trial in which it was used). The mating success for a male was calculated for each day and trial separately because a male’s dominance condition (i.e. bower-holding vs. not bower-holding) might change across days.

To test the prediction that bower-holding males have higher mating success than non-bower holding males, we compared the mating success of males holding bowers and males not holding bowers using unpaired two-tailed t-tests allowing for unequal variance. In order to account for the fact that the tag-readers were not switched on 24 hours a day we repeated this analysis by also including the second most read male as a bower holder. This lead to 6 fish (38-13F6, 2A-01A7, 34-BCEC, 24-9579, 2B-11FC, 1C-04CF, Table S2) being moved from the “no-bower” to the “bower” category.

To assess the effect of bower-holding on mating success, relative to the effects of other male traits, we used linear mixed effects models. Explanatory variables in the models were bower holding (yes or no) and male weight for the analyses on the complete dataset, and bower, male weight, standard length and condition factor for the data set composed of the last three trials. To account for repeated use of some individuals across trials we included individual ID as a random effect. All analyses were done in R (www.r-project.org).

To assess the effect of holding a bower on the mating success of the same male we used a paired two-tailed t-test comparing the mating success between a trial when they held a bower and a trial when they didn’t hold a bower.

### Ethical considerations

All experiments were performed in accordance with relevant guidelines and regulations established by the UK Home Office. All experimental protocols were approved by the UK Home Office (project licence no. 60/3295).

## Additional Information

**How to cite this article**: Magalhaes, I. S. *et al*. Quantifying mating success of territorial males and sneakers in a bower-building cichlid fish. *Sci. Rep.* **7**, 41128; doi: 10.1038/srep41128 (2017).

**Publisher's note:** Springer Nature remains neutral with regard to jurisdictional claims in published maps and institutional affiliations.

## Supplementary Material

Supplementary Material

Supplementary Video S1

## Figures and Tables

**Table 1:** Relative mating success.

Trial	Male ID	SL (cm)	Weight (g)	CF	tag reader number	# offspring	# females	Male MS
1	1B-E516	NA	27.2	NA	1	26	3	0.464
	24-82B0	NA	27.35	NA	2	13	3	0.232
	24-987B	NA	30.61	NA	3	12	3	0.214
	24-90DA	NA	31.26	NA	4B	5	2	0.089
	1B-E918	NA	25.9	NA	4 A	0	0	0.000
2	24-8799	NA	36.05	NA	4 A	25	4	0.658
	24-987B	NA	30.61	NA	1, 2 A, 4B	8	1	0.211
	24-82B0	NA	27.35	NA	3	3	1	0.079
	1B-E918	NA	25.9	NA	no bower	1	1	0.026
	34-BCEC	NA	19.5	NA	1	1	1	0.026
	24-7FDA	NA	19.67	NA	3	0	0	0.000
	2A-01A7	NA	21.88	NA	1, 5	0	0	0.000
	38-13F6	NA	42.23	NA	2B	0	0	0.000
3	24-8799	10.5	31.75	4.822	1	20	5	0.444
	24-7FDA	10.4	31.28	4.878	3	19	4	0.422
	24-987B	9.8	24.1	4.428	no bower	3	1	0.067
	2A-01A7	10	28.88	5.019	2	2	2	0.044
	29-F20E	9.5	27.67	5.54	no bower	1	1	0.022
	2A-F8B7	9.9	31.61	5.648	2	0	0	0.000
	34-BCEC	9.8	24.56	4.513	2	0	0	0.000
4	1C-04CF	9.8	23.37	4.294	2	35	6	0.833
	24-8506	10	25.79	4.482	1, 3	6	2	0.143
	24-9889	8.7	20.39	5.204	no bower	1	1	0.024
	24-9579	10.2	34.51	5.678	1	0	0	0.000
5	1C-04CF	10.4	31.34	4.888	3	25	5	0.431
	1B-F658	10.6	38.09	5.636	3	21	3	0.362
	24-8506	11	37.21	4.971	1	10	2	0.172
	24-8F52	9.4	25.52	5.261	no bower	1	1	0.017
	31-B7F0	10.4	30.5	4.757	no bower	1	1	0.017
	1C-A29A	10.6	30.34	4.489	2	0	0	0.000
	2B-11FC	10.3	31.19	4.996	2	0	0	0.000

Only results for males who fathered offspring and/or owned bowers are shown (see Supplementary Information for detailed results including all males). Within trials males are ordered by decreasing mating success. From left to right: trial number, male ID, standard length (SL) weight and condition factor (CF) of each male; bower ownership (no bower, or tag reader number of bower owned by each male); number of offspring fathered by the male, number of females male mated with; male mating success (Male MS).

**Table 2:** Results of the generalized linear mixed-effects models.

	Value	Std.Error	DF	t-value	p-value
(a) *MS~Bower* + *Weight*					
Intercept	−0.012	0.075	35	−0.160	0.874
**Bower**	**0.17**	**0.034**	**35**	**5.009**	**0.000**
Weight	0.001	0.002	35	0.281	0.780
(b) *MS~Bower* + *SL* + *Weight* + *CF*					
Intercept	1.624	2.721	33	0.597	0.555
**Bower**	**0.204**	**0.05**	**7**	**4.081**	**0.005**
SL	−0.137	0.269	7	−0.511	0.625
Weight	0.018	0.036	7	0.514	0.623
CF	−0.158	0.2188	7	−0.726	0.492

Significant results in bold.
