# Supplementary material for: Quantifying mating success of territorial males and sneakers in a bower-building cichlid fish
Source: Sci Rep. 2017 Jan 27;7:41128. doi: 10.1038/srep41128 (PMC5269580; doi:10.1038/srep41128)
Supplement: Supplementary Material [file srep41128-s1.doc]

Quantifying mating success of territorial males and sneakers in a bower-building cichlid fish

*I.S. Magalhaes ^1,2^, A. M. Smith^1^, D. A. Joyce^1^.

^1^School of Biological, Biomedical and Environmental Sciences, University of Hull, Cottingham Road, Hull, HU6 7RX, U.K.

^2^ University of Nottingham, School of Life Sciences, University Park, Nottingham, NG7 2RD, U.K.

Table of contents for Supporting Information:

Figure S1. Drawing of the location of the reader on the bower………………………………….2

Figure S2. Mating success of males with and without bowers…………………………………..3

Table S1. Experimental schedule………..................................................................................4

Table S2. Summary table for bower ownership assignment………….…………......................5

Table S3. Summary statistics of the microsatellites……………………………………...……….8

Table S4. Summary table of parentage analyses….................................................................9

Table S5. Detailed table of parentage analyses....................................................................10

Table S6. Detailed summary table of mating success of all males........................................15

Legend of video S1 .………………………………………………………………………………..16

Figure S1. Drawing of a bower with the location of the tag-reader on the bower. The tag-reader was connected through a coaxial cable to a tag-logger located outside the water.


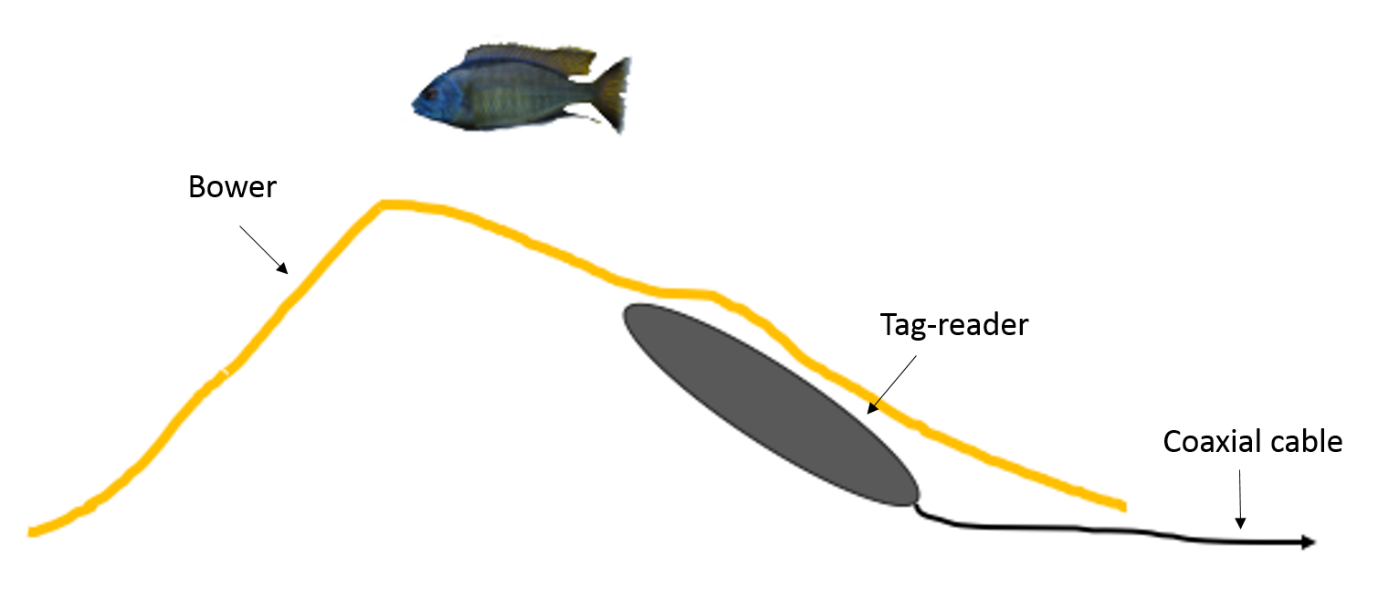


Figure S2. a) Number of males with and without bowers (red and blue respectively) who fathered offspring (lighter coloured section) and who didn’t (darker coloured section) and b) Mean individual relative mating success of bower holding males (blue bar) and non- bower holding males (red bar). Black bars represent standard errors. (unpaired t-test, n1= 25, n2=48, t= 4.044, p (one tailed) <0.001).


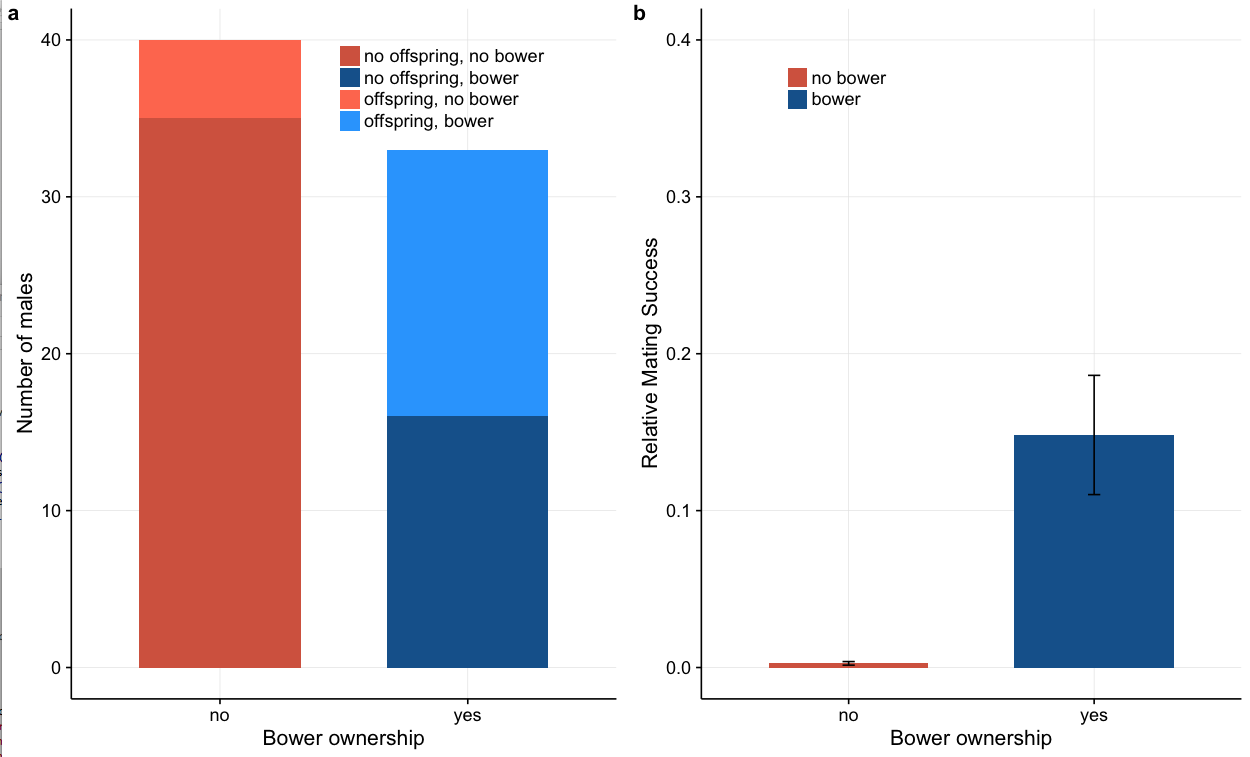


Table S1. Detailed experimental schedule for each of the five trials conducted with dates when males, non-experimental females (“fluffers”) and experimental females were introduced and removed from pond.

| Trial | Dates | Stages of the experiment |
| --- | --- | --- |
| 1 | 20-Jul-09 | Put males and fluffers in pond |
|  | 28-Jul-09 | Removed fluffers, males back in |
|  | 28-Jul-09 | Put 25 females in pond |
|  | 13-Aug-09 | Tag readers in pond, drawing of bower locations |
|  | 18-Aug-09 | Removal of females and males, bowers destroyed |
| 2 | 19-Aug-09 | Put males and fluffers in pond |
|  | 01-Sep-09 | Removed fluffers, males back in |
|  | 03-Sep-09 | Put 25 females in pond |
|  | 20-Sep-09 | Drawing of bower locations |
|  | 23-Sep-09 | Put tag readers in pond |
|  | 29-Sep-09 | Removal of females and males, bowers destroyed |
| 3 | 30-Sep-09 | Put males and fluffers in pond |
|  | 07-Oct-09 | Removed fluffers, males back in |
|  | 07-Oct-09 | Put 25 females in pond |
|  | 20-Oct-09 | Tag readers in pond, drawing of bower locations |
|  | 28-Oct-09 | removal of females and males, bowers destroyed |
| 4 | 20-Jan-10 | Put males and fluffers in pond |
|  | 22-Feb-10 | Removed fluffers, males back in |
|  | 22-Feb-10 | Put 25 females in pond |
|  | 04-Mar-10 | Tag readers in pond, drawing of bower locations |
|  | 16-Mar-10 | Tag readers stopped working |
|  | 18-Mar-10 | Removal of females and males, bowers destroyed |
| 5 | 19-Mar-10 | Put males and fluffers in pond |
|  | 31-Mar-10 | Removed fluffers, males back in |
|  | 31-Mar-10 | Put 25 females in pond |
|  | 21-Apr-10 | Tag readers in pond, drawing of bower locations |
|  | 25-Apr-10 | Tag readers stopped working |
|  | 27-Apr-10 | Removal of females and males, bowers destroyed |

Table S2. Summary table of bower ownership (from left to right): trial number, reader number, date, time of first and last read of the day (Time start, time end), total numbers of tags read, total number of male tags read, tag of dominant male, number of reads of dominant male, percentage of male reads belonging to the dominant male. The following columns are the same but for 2^nd^ and 3^rd^ males with most reads.

| Trial | reader | day | Time | Time | Total # | #reads | TAG of dom. | # reads of dom. male | % of male reads | TAG of 2ndmale | # reads of 2nd male | % of male reads | TAG of 3rd male | #reads of 3rd male | % of male reads |
| --- | --- | --- | --- | --- | --- | --- | --- | --- | --- | --- | --- | --- | --- | --- | --- |
|  |  |  | start | end | reads | males | male |  |  |  |  |  |  |  |  |
| 1 | 5 | 13/08/2009 | 16:51:05 | 18:47:48 | 39 | 39 | DC001BE516 | 39 | 100 |  |  |  |  |  |  |
|  | 5 | 14/08/2009 | 08:58:29 | 14:06:47 | 123 | 123 | DC001BE516 | 122 | 99 |  |  |  |  |  |  |
|  | 5 | 17/08/2009 | 09:37:53 | 13:11:32 | 409 | 284 | DC001BE516 | 228 | 80 |  |  |  |  |  |  |
|  | 5 | 18/08/2009 | 09:07:24 | 09:17:53 | 32 | 15 | DC001BE516 | 8 | 53 | DC003813F6 | 3 | 20 |  |  |  |
| 1 | 6 | 13/08/2009 | 16:17:50 | 18:49:50 | 161 | 161 | DC002482B0 | 161 | 100 |  |  |  |  |  |  |
|  | 6 | 14/08/2009 | 08:57:53 | 14:11:30 | 88 | 88 | DC002482B0 | 88 | 100 |  |  |  |  |  |  |
|  | 6 | 17/08/2009 | 09:52:38 | 13:11:50 | 364 | 277 | DC002482B0 | 173 | 63 | DC00247FDA | 23 | 8 |  |  |  |
|  | 6 | 18/08/2009 | 09:05:31 | 09:17:46 | 48 | 26 | DC002482B0 | 20 | 77 |  |  |  |  |  |  |
| 1 | 7 | 13/08/2009 | 18:22:37 | 18:52:12 | 36 | 36 | DC0024987B | 36 | 100 |  |  |  |  |  |  |
|  | 7 | 14/08/2009 | 08:59:28 | 14:02:47 | 25 | 24 | DC0024987B | 20 | 83 |  |  |  |  |  |  |
|  | 7 | 17/08/2009 | 09:44:55 | 13:11:27 | 361 | 217 | DC0024987B | 97 | 45 | DC002A01A7 | 31 | 14 | DC002490E2 | 28 | 13 |
|  | 7 | 18/08/2009 | 09:07:42 | 09:17:11 | 42 | 27 | DC001A2DBF | 9 | 33 | DC0024987B | 6 | 22 | DC003813F6 | 4 | 15 |
| 1 | 8 | 13/08/2009 | 16:40:44 | 18:38:38 | 28 | 28 | DC001BE918 | 27 | 96 |  |  |  |  |  |  |
|  | 8 | 14/08/2009 | 09:06:03 | 10:21:09 | 14 | 14 | DC001BE918 | 12 | 86 |  |  |  |  |  |  |
|  | 8 | 17/08/2009 | 09:47:13 | 13:08:40 | 344 | 150 | DC002490DA | 98 | 65 | DC002A01A7 | 11 | 7 | DC003813F6 | 8 | 5 |
|  | 8 | 18/08/2009 | 09:05:10 | 09:17:59 | 127 | 17 | DC002490DA | 9 | 53 | DC002A01A7 | 3 | 18 | DC003813F6 | 2 | 12 |
| 2 | 4 | 23/09/2009 | 09:31:43 | 23:45:46 | 537 | 93 | DC002A01A7 | 50 | 54 | DC0024987B | 17 | 18 |  |  |  |
|  | 4 | 24/09/2009 | 00:02:44 | 23:58:39 | 2331 | 617 | DC0034BCEC | 169 | 27 | DC001BE404 | 147 | 24 | DC002490E2 | 140 | 23 |
|  | 4 | 25/09/2009 | 00:04:11 | 11:43:35 | 1898 | 1129 | DC0024987B | 791 | 70 | DC0034BCEC | 176 | 16 |  |  |  |
|  | 4 | 28/09/2009 | 11:34:17 | 13:16:14 | 147 | 66 | DC0024987B | 5 0 | 76 |  |  |  |  |  |  |
| 2 | 5 | 23/09/2009 | 09:54:41 | 23:56:03 | 857 | 755 | DC0024987B | 696 | 92 |  |  |  |  |  |  |
|  | 5 | 24/09/2009 | 00:07:00 | 23:29:53 | 2574 | 1936 | DC0024987B | 1884 | 97 |  |  |  |  |  |  |
|  | 5 | 25/09/2009 | 00:04:31 | 11:44:08 | 1171 | 540 | DC0024987B | 220 | 41 | DC0034BCEC | 133 | 25 | DC002AF8B7 | 92 | 17 |
|  | 5 | 28/09/2009 | 11:34:23 | 13:02:50 | 261 | 150 | DC003813F6 | 128 | 85 |  |  |  |  |  |  |
| 2 | 6 | 23/09/2009 | 09:51:10 | 23:58:56 | 566 | 482 | DC002482B0 | 442 | 92 |  |  |  |  |  |  |
|  | 6 | 24/09/2009 | 00:00:56 | 23:49:49 | 3000 | 1753 | DC002482B0 | 1700 | 97 |  |  |  |  |  |  |
|  | 6 | 25/09/2009 | 00:03:24 | 11:43:39 | 1419 | 1224 | DC002482B0 | 1177 | 96 |  |  |  |  |  |  |
|  | 6 | 28/09/2009 | 11:34:27 | 13:18:13 | 207 | 178 | DC00247FDA | 178 | 100 |  |  |  |  |  |  |
| 2 | 7 | 23/09/2009 | 10:55:39 | 23:41:32 | 63 | 34 | DC00248799 | 20 | 59 | DC002A01A7 | 13 | 38 |  |  |  |
|  | 7 | 24/09/2009 | 00:31:45 | 23:59:42 | 1355 | 313 | DC00248799 | 259 | 83 |  |  |  |  |  |  |
|  | 7 | 25/09/2009 | 00:33:59 | 11:43:12 | 337 | 257 | DC00248799 | 158 | 62 | DC00247FDA | 43 | 17 |  |  |  |
|  | 7 | 28/09/2009 | 11:43:14 | 13:13:57 | 58 | 19 | DC0024987B | 8 | 42 | DC00248A4E | 7 | 37 |  |  |  |
| 2 | 8 | 23/09/2009 | 09:30:11 | 23:54:18 | 392 | 264 | DC002A01A7 | 259 | 98 |  |  |  |  |  |  |
|  | 8 | 24/09/2009 | 00:24:02 | 23:41:22 | 2060 | 1952 | DC002A01A7 | 1941 | 99 |  |  |  |  |  |  |
|  | 8 | 25/09/2009 | 00:07:28 | 11:43:57 | 1285 | 1241 | DC002A01A7 | 1224 | 99 |  |  |  |  |  |  |
|  | 8 | 28/09/2009 | 11:41:53 | 13:17:18 | 76 | 74 | DC002A01A7 | 73 | 99 |  |  |  |  |  |  |
| 3 | 6 | 20/10/2009 | 10:43:57 | 23:22:46 | 381 | 333 | DC00248799 | 237 | 71 |  |  |  |  |  |  |
|  | 6 | 21/10/2009 | 00:27:54 | 23:36:15 | 825 | 697 | DC00248799 | 631 | 91 |  |  |  |  |  |  |
|  | 6 | 22/10/2009 | 00:36:19 | 23:43:56 | 1212 | 862 | DC00248799 | 789 | 92 |  |  |  |  |  |  |
|  | 6 | 23/10/2009 | 00:12:42 | 12:43:08 | 684 | 586 | DC00248799 | 526 | 90 |  |  |  |  |  |  |
|  | 6 | 27/10/2009 | 10:26:40 | 23:59:08 | 1789 | 725 | DC00248799 | 604 | 83 |  |  |  |  |  |  |
|  | 6 | 28/10/2009 | 00:03:59 | 10:00:57 | 1187 | 1013 | DC00248799 | 969 | 96 |  |  |  |  |  |  |
| 3 | 7 | 20/10/2009 | 10:44:47 | 23:48:33 | 760 | 617 | DC002A01A7 | 546 | 89 |  |  |  |  |  |  |
|  | 7 | 21/10/2009 | 00:04:31 | 23:53:58 | 2284 | 1062 | DC002A01A7 | 653 | 62 |  |  |  |  |  |  |
|  | 7 | 22/10/2009 | 00:02:09 | 23:59:24 | 4913 | 1478 | DC0034BCEC | 306 | 21 | DC002A01A7 | 246 | 17 | DC002490E2 | 334 | 23 |
|  | 7 | 23/10/2009 | 00:00:08 | 12:44:15 | 7483 | 1699 | DC0034BCEC | 712 | 42 | DC002490E2 | 414 | 24 | DC001BE516 | 92 | 5 |
|  | 7 | 27/10/2009 | 10:27:20 | 23:59:13 | 6662 | 881 | DC002AF8B7 | 222 | 25 | DC002490E2 | 155 | 18 | DC003813F6 | 142 | 16 |
|  | 7 | 28/10/2009 | 00:00:04 | 10:01:14 | 10140 | 1086 | DC002AF8B7 | 191 | 18 | DC0034BCEC | 182 | 17 | DC003813F6 | 168 | 16 |
| 3 | 8 | 20/10/2009 | 11:31:40 | 23:52:51 | 560 | 468 | DC00247FDA | 436 | 93 |  |  |  |  |  |  |
|  | 8 | 21/10/2009 | 00:04:41 | 23:55:22 | 1181 | 1016 | DC00247FDA | 964 | 95 |  |  |  |  |  |  |
|  | 8 | 22/10/2009 | 00:02:20 | 23:58:45 | 1367 | 1147 | DC00247FDA | 1071 | 93 |  |  |  |  |  |  |
|  | 8 | 23/10/2009 | 00:00:37 | 12:41:24 | 515 | 428 | DC00247FDA | 404 | 94 |  |  |  |  |  |  |
|  | 8 | 27/10/2009 | 10:27:46 | 23:54:19 | 1206 | 724 | DC00247FDA | 657 | 91 |  |  |  |  |  |  |
|  | 8 | 28/10/2009 | 00:00:33 | 09:55:59 | 652 | 513 | DC00247FDA | 487 | 95 |  |  |  |  |  |  |
| 4 | 1 | 04/03/2010 | 13:49:13 | 23:50:08 | 324 | 160 | DC00248506 | 50 | 31 | DC00249579 | 40 | 25 | DC002478D0 | 39 | 24 |
|  | 1 | 09/03/2010 | 10:25:54 | 11:13:21 | 181 | 60 | DC00249579 | 43 | 72 | DC002B11FC | 3 | 5 |  |  |  |
|  | 1 | 16/03/2010 | 10:16:50 | 18:23:12 | 629 | 374 | DC00249579 | 258 | 69 |  |  |  |  |  |  |
| 4 | 2 | 04/03/2010 | 13:41:52 | 23:59:59 | 29277 | 121 | DC001C04CF | 94 | 78 |  |  |  |  |  |  |
|  | 2 | 05/03/2010 | 00:00:01 | 00:33:46 | 1500 | 52 | DC001C04CF | 44 | 85 |  |  |  |  |  |  |
|  | 2 | 09/03/2010 | 10:25:52 | 11:13:31 | 2292 | 10 | DC001C04CF | 4 | 40 | DC0031B7F0 | 4 | 40 |  |  |  |
|  | 2 | 16/03/2010 | 10:16:49 | 18:23:39 | 24590 | 24 | DC001C04CF | 21 | 88 |  |  |  |  |  |  |
| 4 | 7 | 16/03/2010 | 11:56:09 | 18:21:06 | 764 | 648 | DC00248506 | 638 | 99 |  |  |  |  |  |  |
| 5 | 1 | 23/04/2010 | 16:23:12 | 23:58:02 | 199 | 90 | DC00248506 | 86 | 96 |  |  |  |  |  |  |
|  | 1 | 24/04/2010 | 00:03:15 | 08:38:37 | 267 | 208 | DC00248506 | 191 | 92 |  |  |  |  |  |  |
| 5 | 4 | 23/04/2010 | 16:55:15 | 23:59:34 | 2276 | 239 | DC001CA29A | 103 | 43 | DC002A0C48 | 76 | 32 | DC002A0855 | 36 | 15 |
|  | 4 | 24/04/2010 | 00:00:26 | 08:39:32 | 8370 | 177 | DC002B11FC | 84 | 48 | DC001CA29A | 22 | 12 | DC002A0C48 | 20 | 11 |
| 5 | 8 | 22/04/2010 | 15:26:09 | 23:59:04 | 238 | 183 | DC001BF658 | 127 | 69 | DC001CA29A | 20 | 11 |  |  |  |
|  | 8 | 23/04/2010 | 00:32:18 | 23:24:27 | 757 | 558 | DC001BF658 | 377 | 68 | DC001C04CF | 72 | 13 | DC001CA29A | 56 | 10 |
|  | 8 | 24/04/2010 | 00:23:07 | 08:41:27 | 247 | 218 | DC001C04CF | 203 | 93 |  |  |  |  |  |  |

Table S3. Summary statistics for the 4 microsatellites used for parentage assignment: number of alleles (A), allelic richness (AR) and observed and expected heterozygosity.

|  | ppun5 | ppun7 | ppun21 | TMOM5 |
| --- | --- | --- | --- | --- |
| A | 12 | 18 | 17 | 16 |
| AR | 11.988 | 17.994 | 16.982 | 16 |
| HO | 0.821 | 0.748 | 0.873 | 0.762 |
| HE | 0.819 | 0.797 | 0.861 | 0.836 |

Table S4. Summary table of paternity analyses: Confidence level: strict, relaxed or unassigned **(Level**), confidence level expressed in percentage terms **(Confidence (%)),** critical value of Delta for this level of confidence estimated by the simulation **(Critical Delta)**, observed number of offspring for which parentage is assigned using this critical value of Delta (the expected number of parentage assignments predicted by the simulation is shown in brackets) (**Assignments),** observed percentage of offspring for which parentage is assigned (the expected percentage of parentage assignments predicted by the simulation is shown in brackets) (**Assignment Rate).**

| Level | Confidence | Critical Delta | Assignments | Assignment rate |
| --- | --- | --- | --- | --- |
|  | (%) |  |  |  |
| *Trial 1* |  |  |  |  |
| Strict | 95 | 2.1 | 49(25) | 84% (43%) |
| Relaxed | 80 | 0.42 | 56(45) | 97% (77%) |
| Unassigned | |  | 2(13) | 3% (23%) |
| Total |  |  | 58(58) | 100% (100%) |
| *Trial 2* |  |  |  |  |
| Strict | 95 | 0.69 | 31(30) | 0.76(74%) |
| Relaxed | 80 | 0 | 38(39) | 0.93(96%) |
| Unassigned | |  | 3(2) | 7% (4%) |
| Total |  |  | 41(41) | 100% (100%) |
| *Trial 3* |  |  |  |  |
| Strict | 95 | 1.37 | 34(28) | 76% (62%) |
| Relaxed | 80 | 0 | 45(42) | 100% (93%) |
| Unassigned | |  | 0(3) | 0% (7%) |
| Total |  |  | 45(45) | 100% (100%) |
| *Trial 4* |  |  |  |  |
| Strict | 95 | 1.86 | 42(36) | 76% (65%) |
| Relaxed | 80 | 0 | 45(46) | 82% (83%) |
| Unassigned | |  | 10(9) | 18% (17%) |
| Total |  |  | 55(55) | 100% (100%) |
| *Trial 5* |  |  |  |  |
| Strict | 95 | 1 | 57(47) | 93% (76%) |
| Relaxed | 80 | 0 | 58(53) | 95% (88%) |
| Unassigned | |  | 3(8) | 5% (12%) |
| Total |  |  | 61(61) | 100% (100%) |

Table S5. Summary results of paternity assignment analyses done in CERVUS (from left to right): name of the offspring being tested (**Offspring ID),**  name of known parent **(Mother ID),** log-likelihood ratio for a parent-offspring relationship between the known parent and the offspring (**Pair LOD score),**  name of the most likely candidate parent (**Candidate father ID**), log-likelihood ratio and Delta for a parent-offspring relationship between the most likely candidate parent and the offspring (**Pair LOD score, Pair Delta),** the log-likelihood ratio and Delta value for a parent-offspring relationship between the candidate parent and the offspring given the known parent (**Trio LOD score, Trio Delta),** confidence of parentage assignment taking in account known parent (**Trio confidence):** * for strict confidence or + for relaxed confidence. - is shown for a most likely candidate parent not assigned parentage. If the candidate parent is not most likely, this column will be blank. The number of loci typed in the known parent was always 4, and so was the number of loci typed in the offspring. There were no mismatches between the two. The number of loci typed in both the offspring and the candidate parent was always 4. There were zero mismatches between the loci of the candidate parent and those of the offspring and also between offspring, known parent and candidate parent**.**

| Offspring ID | Mother ID | Pair LOD score | Candidate father ID | Pair LOD score | Pair Delta | Pair confidence | Trio LOD score | Trio Delta | Trio confidence |
| --- | --- | --- | --- | --- | --- | --- | --- | --- | --- |
|
| T1F11.1 | 29-EE70 | 3.53 | 1B-E516 | 1.19 | 1.19 | + | 3.25 | 3.25 | * |
| T1F11.2 | 29-EE70 | 3.29 | 1B-E516 | 0.38 | 0.38 | - | 1.76 | 1.76 | + |
| T1F11.10 | 29-EE70 | 3.29 | 1B-E516 | 1.12 | 1.12 | + | 2.50 | 2.50 | * |
| T1F11.4 | 29-EE70 | 3.22 | 1B-E516 | 1.51 | 0.51 | - | 4.06 | 4.06 | * |
| T1F11.5 | 29-EE70 | 3.61 | 1B-E516 | -0.30 | 0.00 |  | 1.67 | 1.67 | + |
| T1F11.6 | 29-EE70 | 3.11 | 1B-E516 | 1.84 | 0.88 | - | 4.07 | 4.01 | * |
| T1F11.3 | 29-EE70 | 5.38 | 1B-E516 | 0.45 | 0.45 | - | 2.44 | 2.44 | * |
| T1F11.7 | 29-EE70 | 6.07 | 1B-E516 | -0.97 | 0.00 |  | 1.61 | 1.61 | + |
| T1F11.9 | 29-EE70 | 4.64 | 1B-E516 | 1.84 | 0.88 | - | 4.08 | 4.01 | * |
| T1F2.1 | 37-F360 | 3.57 | 24-82B0 | 1.99 | 1.53 | + | 2.38 | 2.38 | * |
| T1F2.2 | 37-F360 | 2.55 | 24-987B | 1.19 | 0.00 |  | 3.77 | 3.77 | * |
| T1F2.3 | 37-F360 | 1.28 | 24-82B0 | 3.33 | 2.75 | * | 3.64 | 3.64 | * |
| T1F2.4 | 37-F360 | 1.96 | 24-82B0 | 2.02 | 1.26 | + | 2.52 | 2.52 | * |
| T1F2.5 | 37-F360 | 3.52 | 24-82B0 | 3.20 | 3.20 | * | 3.89 | 3.89 | * |
| T1F3.1 | 24-7434 | 4.06 | 24-987B | 3.49 | 3.49 | * | 4.94 | 4.94 | * |
| T1F3.10 | 24-7434 | 0.52 | 24-987B | 3.22 | 1.14 | + | 4.07 | 4.07 | * |
| T1F3.2 | 24-7434 | 3.56 | 24-987B | 3.28 | 3.28 | * | 4.45 | 4.45 | * |
| T1F3.3 | 24-7434 | 2.77 | 24-987B | 3.12 | 2.40 | + | 4.35 | 4.35 | * |
| T1F3.4 | 24-7434 | 4.67 | 24-987B | 2.97 | 2.97 | * | 4.94 | 4.94 | * |
| T1F3.5 | 24-7434 | 3.04 | 24-90DA | 1.21 | 1.21 | + | 1.93 | 1.93 | + |
| T1F3.6 | 24-7434 | 4.31 | 24-987B | 2.60 | 2.40 | + | 4.35 | 4.35 | * |
| T1F3.7 | 24-7434 | 2.25 | 24-987B | 2.34 | 1.14 | + | 3.53 | 3.53 | * |
| T1F3.8 | 24-7434 | 3.75 | 24-987B | 2.93 | 2.93 | * | 3.90 | 3.90 | * |
| T1F3.9 | 24-7434 | 2.16 | 24-987B | 3.98 | 3.98 | * | 4.67 | 4.67 | * |
| T1F4.1 | 2B-0F23 | 5.21 | 1B-E516 | 1.70 | 1.70 | + | 4.45 | 4.45 | * |
| T1F4.10 | 2B-0F23 | 5.21 | 1B-E516 | 0.59 | 0.59 | - | 3.33 | 3.33 | * |
| T1F4.11 | 2B-0F23 | 3.95 | 1B-E516 | 0.96 | 0.96 | - | 3.26 | 3.26 | * |
| T1F4.12 | 2B-0F23 | 3.62 | 1B-E516 | 0.46 | 0.46 | - | 2.51 | 2.51 | * |
| T1F4.2 | 2B-0F23 | 4.92 | 1B-E516 | 0.46 | 0.42 | - | 2.51 | 2.51 | * |
| T1F4.3 | 2B-0F23 | 4.52 | 1B-E516 | -0.09 | 0.00 |  | 1.76 | 1.76 | + |
| T1F4.4 | 2B-0F23 | 3.62 | 1B-E516 | 2.01 | 2.01 | + | 4.08 | 4.08 | * |
| T1F4.5 | 2B-0F23 | 3.95 | 1B-E516 | 1.33 | 1.33 | + | 3.64 | 3.64 | * |
| T1F4.6 | 2B-0F23 | 3.91 | 1B-E516 | -0.97 | 0.00 |  | 1.75 | 1.75 | + |
| T1F4.7 | 2B-0F23 | 3.46 | 1B-E516 | 2.58 | 2.58 | + | 4.45 | 4.45 | * |
| T1F4.8 | 2B-0F23 | 3.46 | 1B-E516 | 1.03 | 1.03 | - | 2.87 | 2.87 | * |
| T1F4.9 | 2B-0F23 | 3.74 | 1B-E516 | 2.38 | 2.38 | + | 4.45 | 4.45 | * |
| T1F6.1 | 2A-0B8C | 2.07 | 24-987B | 2.24 | 2.10 | + | 4.23 | 4.23 | * |
| T1F6.2 | 2A-0B8C | 1.71 | 24-82B0 | 1.25 | 0.00 |  | 3.30 | 3.30 | * |
| T1F6.3 | 2A-0B8C | 2.22 | 24-82B0 | 3.63 | 3.63 | * | 5.00 | 5.00 | * |
| T1F6.4 | 2A-0B8C | 1.23 | 24-987B | 2.04 | 1.26 | + | 3.93 | 3.93 | * |
| T1F6.5 | 2A-0B8C | 2.13 | 24-82B0 | 3.10 | 2.27 | + | 3.31 | 3.31 | * |
| T1F6.6 | 2A-0B8C | 1.91 | 24-82B0 | 2.47 | 1.89 | + | 2.67 | 2.67 | * |
| T1F6.7 | 2A-0B8C | 2.34 | 24-82B0 | 2.67 | 2.02 | + | 2.84 | 2.84 | * |
| T1F6.8 | 2A-0B8C | 2.11 | 24-82B0 | 2.67 | 1.89 | + | 2.84 | 2.84 | * |
| T1F6.9 | 2A-0B8C | 2.15 | 24-82B0 | 0.82 | 0.00 |  | 2.83 | 2.83 | * |
| T1F7.3 | 1B-FF97 | 3.86 | 1B-E516 | -0.23 | 0.00 |  | 2.49 | 2.49 | * |
| T1F7.4 | 1B-FF97 | 3.30 | 1B-E516 | 0.51 | 0.00 |  | 3.24 | 3.24 | * |
| T1F7.1 | 1B-FF97 | 3.28 | 1B-E516 | -0.22 | 0.00 |  | 2.50 | 2.50 | * |
| T1F7.2 | 1B-FF97 | 3.99 | 24-82B0 | 1.34 | 0.00 |  | 2.19 | 2.19 | * |
| T1F7.5 | 1B-FF97 | 3.34 | 1B-E516 | 0.96 | 0.00 |  | 3.70 | 3.70 | * |
| T1F7.6 | 1B-FF97 | 2.02 | 1B-E516 | 1.70 | 0.00 |  | 4.43 | 4.43 | * |
| T1F7.7 | 1B-FF97 | 4.17 | 24-82B0 | 2.32 | 0.00 |  | 4.16 | 4.00 | * |
| T1F8.1 | 2A-110D | 1.98 | 24-90DA | 1.24 | 0.00 |  | 2.34 | 0.68 | + |
| T1F8.2 | 2A-110D | 2.29 | 24-90DA | 1.56 | 1.18 | + | 2.93 | 2.93 | * |
| T1F8.3 | 2A-110D | 4.98 | 24-90DA | 0.88 | 0.88 | - | 2.71 | 2.71 | * |
| T1F8.4 | 2A-110D | 3.81 | 24-90DA | 1.59 | 0.00 |  | 2.71 | 2.71 | * |
| T2F1.1 | 2A-0D1F | 2.84 | 24-8799 | 0.70 | 0.65 | + | 2.74 | 0.68 | + |
| T2F1.2 | 2A-0D1F | 0.54 | 24-8799 | 1.02 | 0.03 | - | 2.38 | 2.38 | * |
| T2F1.3 | 2A-0D1F | 2.84 | 24-8799 | 1.21 | 1.13 | + | 3.26 | 3.26 | * |
| T2F2.3 | 2A-FE66 | 2.68 | 24-82B0 | 1.24 | 1.21 | + | 3.00 | 3.00 | * |
| T2F2.4 | 2A-FE66 | 6.28 | 24-82B0 | 4.27 | 4.27 | * | 6.91 | 6.91 | * |
| T2F2.5 | 2A-FE66 | 4.68 | 24-82B0 | 3.18 | 3.18 | * | 5.12 | 5.12 | * |
| T2F4.1 | 24-7E2C | 2.59 | 24-987B | 0.79 | 0.79 | + | 2.36 | 2.36 | * |
| T2F4.10 | 24-7E2C | 5.64 | 24-987B | 1.21 | 1.21 | + | 3.06 | 3.06 | * |
| T2F4.11 | 24-7E2C | 4.66 | 24-987B | 0.74 | 0.00 |  | 2.14 | 2.14 | * |
| T2F4.2 | 24-7E2C | 3.46 | 24-987B | 3.79 | 3.79 | * | 5.38 | 5.38 | * |
| T2F4.3 | 24-7E2C | 4.26 | 24-987B | -0.17 | 0.00 |  | 1.67 | 1.67 | * |
| T2F4.4 | 24-7E2C | 3.55 | 24-987B | 0.32 | 0.00 |  | 2.36 | 2.36 | * |
| T2F4.5 | 24-7E2C | 2.08 | 24-987B | 0.79 | 0.00 |  | 2.36 | 2.36 | * |
| T2F4.7 | 24-7E2C | 1.40 | 34-BCEC | 7.31 | 3.18 | * | 10.10 | 9.08 | * |
| T2F4.8 | 24-7E2C | 4.24 | 1B-E918 | 3.71 | 1.58 | + | 5.10 | 5.10 | * |
| T2F4.9 | 24-7E2C | 2.59 | 24-987B | 2.41 | 2.41 | * | 4.00 | 4.00 | * |
| T2F6.1 | 29-EE70 | 3.65 | 24-8799 | -0.14 | 0.00 |  | 1.67 | 0.68 | + |
| T2F6.3 | 29-EE70 | 1.69 | 24-8799 | 0.82 | 0.04 | - | 2.30 | 2.30 | * |
| T2F6.4 | 29-EE70 | 3.84 | 24-8799 | 0.54 | 0.54 | + | 1.76 | 0.68 | + |
| T2F6.5 | 29-EE70 | 2.99 | 24-8799 | 0.97 | 0.97 | + | 3.10 | 3.10 | * |
| T2F6.7 | 29-EE70 | 3.84 | 24-8799 | 0.54 | 0.54 | + | 1.76 | 0.68 | + |
| T2F6.8 | 29-EE70 | 2.99 | 24-8799 | 1.00 | 1.00 | + | 3.13 | 3.13 | * |
| T2F7.1 | 38-0BE7 | 2.18 | 24-8799 | 1.24 | 1.24 | + | 1.92 | 1.37 | * |
| T2F7.3 | 38-0BE7 | 0.60 | 24-8799 | 1.25 | 0.65 | + | 1.93 | 1.93 | * |
| T2F7.4 | 38-0BE7 | 3.54 | 24-8799 | 1.06 | 1.06 | + | 2.29 | 2.29 | * |
| T2F7.5 | 38-0BE7 | 1.93 | 24-8799 | 1.05 | 0.00 |  | 2.26 | 2.26 | * |
| T2F7.6 | 38-0BE7 | 1.97 | 24-8799 | 0.57 | 0.00 |  | 1.80 | 1.80 | * |
| T2F7.7 | 38-0BE7 | 1.79 | 24-8799 | 1.39 | 0.27 | - | 2.76 | 1.37 | * |
| T2F7.8 | 38-0BE7 | 0.59 | 24-8799 | 1.24 | 1.24 | + | 1.92 | 1.37 | * |
| T2F8.1 | 34-C4F5 | 2.21 | 24-8799 | 1.02 | 1.02 | + | 2.39 | 2.39 | * |
| T2F8.10 | 34-C4F5 | 2.00 | 24-8799 | 1.04 | 0.39 | - | 2.40 | 2.40 | * |
| T2F8.11 | 34-C4F5 | 3.51 | 24-8799 | 0.54 | 0.54 | + | 1.91 | 0.69 | + |
| T2F8.2 | 34-C4F5 | 3.32 | 24-8799 | 1.05 | 1.05 | + | 2.43 | 2.43 | * |
| T2F8.3 | 34-C4F5 | 2.21 | 24-8799 | 1.02 | 1.02 | + | 2.39 | 2.39 | * |
| T2F8.4 | 34-C4F5 | 3.54 | 24-8799 | 1.05 | 1.05 | + | 2.43 | 2.43 | * |
| T2F8.5 | 34-C4F5 | 2.21 | 24-8799 | 0.54 | 0.54 | + | 1.90 | 0.69 | + |
| T2F8.8 | 34-C4F5 | 3.51 | 24-8799 | 0.54 | 0.54 | + | 1.91 | 0.69 | + |
| T2F8.9 | 34-C4F5 | 2.22 | 24-8799 | 0.55 | 0.55 | + | 1.92 | 1.37 | * |
| T3F2.1 | 24-7FF4 | 1.27 | 24-8799 | 1.95 | 0.68 | - | 2.64 | 2.64 | * |
| T3F2.2 | 24-7FF4 | 0.94 | 24-8799 | 1.40 | 0.46 | - | 2.15 | 2.15 | * |
| T3F2.3 | 24-7FF4 | -0.11 | 29-F20E | 1.25 | 0.29 | - | 1.65 | 0.00 | + |
| T3F2.4 | 24-7FF4 | 0.83 | 2A-01A7 | 1.99 | 1.16 | + | 2.68 | 2.68 | * |
| T3F2.5 | 24-7FF4 | 1.64 | 24-8799 | 2.11 | 0.46 | - | 3.44 | 3.44 | * |
| T3F3.1 | 1B-FF97 | 1.31 | 24-7FDA | 1.13 | 0.81 | + | 2.25 | 2.25 | * |
| T3F3.2 | 1B-FF97 | 1.07 | 24-8799 | 0.57 | 0.57 | - | 0.90 | 0.68 | + |
| T3F3.3 | 1B-FF97 | 3.21 | 24-7FDA | 1.01 | 0.00 |  | 3.13 | 3.13 | * |
| T3F3.4 | 1B-FF97 | 3.09 | 24-7FDA | 0.28 | 0.28 | - | 2.52 | 2.52 | * |
| T3F3.5 | 1B-FF97 | 3.64 | 24-7FDA | 0.73 | 0.73 | - | 2.67 | 2.67 | * |
| T3F3.6 | 1B-FF97 | 2.94 | 24-7FDA | 0.01 | 0.01 | - | 1.56 | 1.56 | * |
| T3F3.7 | 1B-FF97 | -0.79 | 24-7FDA | 1.62 | 0.81 | + | 2.24 | 2.24 | * |
| T3F3.8 | 1B-FF97 | 1.31 | 24-7FDA | 1.13 | 0.81 | + | 2.25 | 2.25 | * |
| T3F4.1 | 29-F3C5 | 3.83 | 24-8799 | -0.79 | 0.00 |  | 0.52 | 0.52 | + |
| T3F4.2 | 29-F3C5 | 2.60 | 24-8799 | 1.42 | 1.28 | + | 3.48 | 3.48 | * |
| T3F4.3 | 29-F3C5 | -0.79 | 24-987B | 2.73 | 1.33 | + | 4.45 | 4.45 | * |
| T3F4.4 | 29-F3C5 | 2.45 | 24-8799 | 1.50 | 1.50 | + | 3.05 | 3.05 | * |
| T3F4.5 | 29-F3C5 | 2.64 | 24-8799 | 0.48 | 0.48 | - | 1.82 | 1.82 | * |
| T3F4.6 | 29-F3C5 | 2.29 | 24-8799 | 0.57 | 0.57 | - | 1.06 | 0.68 | + |
| T3F4.7 | 29-F3C5 | 2.45 | 24-8799 | 0.84 | 0.84 | + | 2.02 | 2.02 | * |
| T3F4.8 | 29-F3C5 | 1.07 | 24-987B | 1.74 | 0.80 | + | 4.26 | 4.26 | * |
| T3F4.9 | 29-F3C5 | 0.24 | 24-8799 | 2.04 | 2.04 | + | 3.46 | 3.46 | * |
| T3F4.10 | 29-F3C5 | 1.07 | 24-8799 | 1.72 | 1.57 | + | 3.47 | 3.47 | * |
| T3F4.11 | 29-F3C5 | 2.64 | 24-8799 | 0.48 | 0.48 | - | 1.82 | 1.82 | * |
| T3F4.12 | 29-F3C5 | 3.32 | 24-8799 | -0.11 | 0.00 |  | 0.87 | 0.68 | + |
| T3F4.13 | 29-F3C5 | 2.80 | 24-8799 | -0.11 | 0.00 |  | 0.71 | 0.68 | + |
| T3F4.14 | 29-F3C5 | 1.76 | 24-8799 | 1.08 | 1.08 | + | 2.45 | 2.45 | * |
| T3F4.15 | 29-F3C5 | 2.29 | 24-8799 | 0.57 | 0.57 | - | 1.06 | 0.68 | + |
| T3F4.16 | 29-F3C5 | 0.04 | 24-987B | 2.41 | 0.80 | + | 4.46 | 4.46 | * |
| T3F5.2 | 24-81E9 | 4.20 | 2A-01A7 | 6.55 | 6.17 | * | 8.37 | 7.71 | * |
| T3F5.6 | 24-81E9 | 2.31 | 24-8799 | 0.13 | 0.13 | - | 1.25 | 0.69 | + |
| T3F5.7 | 24-81E9 | 4.43 | 24-7FDA | -0.76 | 0.00 |  | 1.41 | 1.41 | * |
| T3F5.1 | 24-81E9 | 4.66 | 24-7FDA | 1.95 | 1.95 | + | 3.90 | 3.90 | * |
| T3F5.8 | 24-81E9 | 2.99 | 24-7FDA | 3.07 | 0.09 | - | 4.46 | 4.46 | * |
| T3F5.9 | 24-81E9 | 3.09 | 24-7FDA | 1.01 | 0.00 |  | 2.01 | 2.01 | * |
| T3F5.4 | 24-81E9 | 4.59 | 24-7FDA | 1.17 | 1.17 | + | 3.22 | 3.22 | * |
| T3F6.2 | 2B-0FCB | 1.83 | 24-8799 | 1.00 | 0.67 | - | 1.68 | 0.69 | + |
| T3F6.3 | 2B-0FCB | 2.95 | 24-7FDA | 1.62 | 1.62 | + | 2.22 | 2.22 | * |
| T3F6.6 | 2B-0FCB | 1.42 | 24-7FDA | 1.50 | 1.50 | + | 1.82 | 1.82 | * |
| T3F6.4 | 2B-0FCB | 4.35 | 24-7FDA | -0.47 | 0.00 |  | 1.87 | 1.87 | * |
| T3F6.5 | 2B-0FCB | 2.10 | 24-7FDA | 1.72 | 1.72 | + | 2.76 | 2.76 | * |
| T3F6.7 | 2B-0FCB | 3.47 | 24-8799 | -0.79 | 0.00 |  | 1.12 | 0.67 | + |
| T3F6.8 | 2B-0FCB | 3.98 | 24-7FDA | 0.28 | 0.28 | - | 1.24 | 1.24 | + |
| T3F6.9 | 2B-0FCB | 2.66 | 24-7FDA | 1.77 | 1.77 | + | 2.78 | 2.78 | * |
| T3F7.1 | 34-C8C7 | 6.46 | 24-7FDA | 0.16 | 0.16 | - | 1.50 | 1.50 | * |
| T4F16.1 | 29-F4F3 | 2.30 | 1C-04CF | 2.51 | 2.51 | + | 4.58 | 4.58 | * |
| T4F16.2 | 29-F4F3 | 1.52 | 1C-04CF | 1.98 | 1.98 | + | 3.56 | 3.56 | * |
| T4F16.3 | 29-F4F3 | 1.76 | 1C-04CF | 2.51 | 2.51 | + | 4.57 | 4.57 | * |
| T4F16.4 | 29-F4F3 | 3.49 | 1C-04CF | 2.42 | 2.42 | + | 4.01 | 4.01 | * |
| T4F16.5 | 29-F4F3 | 2.47 | 1C-04CF | 1.34 | 0.00 |  | 2.71 | 2.71 | * |
| T4F17.1 | 2A-FE66 | 4.28 | 1C-04CF | -0.12 | 0.00 |  | 1.78 | 1.78 | + |
| T4F17.2 | 2A-FE66 | 2.36 | 1C-04CF | 2.13 | 2.13 | + | 3.93 | 3.93 | * |
| T4F17.3 | 2A-FE66 | 3.28 | 1C-04CF | 0.73 | 0.07 | - | 2.89 | 2.89 | * |
| T4F17.4 | 2A-FE66 | 2.35 | 1C-04CF | 0.94 | 0.94 | + | 2.57 | 2.57 | * |
| T4F17.5 | 2A-FE66 | 3.80 | 24-9889 | 2.13 | 0.00 |  | 2.97 | 2.97 | * |
| T4F17.6 | 2A-FE66 | 2.35 | 1C-04CF | 0.94 | 0.94 | + | 2.57 | 2.57 | * |
| T4F19.3 | 24-75AB | 3.70 | 1C-04CF | 0.52 | 0.00 |  | 1.88 | 1.88 | * |
| T4F19.4 | 24-75AB | 2.20 | 1C-04CF | 1.71 | 0.00 |  | 2.50 | 2.50 | * |
| T4F20.3 | 24-8E83 | 6.10 | 24-8506 | 3.83 | 3.83 | * | 6.61 | 6.61 | * |
| T4F20.4 | 24-8E83 | 6.27 | 24-8506 | 3.04 | 3.04 | + | 5.80 | 5.80 | * |
| T4F20.5 | 24-8E83 | 6.27 | 24-8506 | 3.04 | 3.04 | + | 5.80 | 5.80 | * |
| T4F20.6 | 24-8E83 | 5.00 | 24-8506 | 3.04 | 3.04 | + | 5.80 | 5.80 | * |
| T4F21.1 | 2B-156D | 4.34 | 1C-04CF | 1.74 | 1.74 | + | 3.80 | 3.80 | * |
| T4F21.10 | 2B-156D | 0.64 | 1C-04CF | 1.45 | 1.45 | + | 3.92 | 3.92 | * |
| T4F21.12 | 2B-156D | 3.21 | 1C-04CF | 1.74 | 1.74 | + | 3.80 | 3.80 | * |
| T4F21.13 | 2B-156D | 3.59 | 1C-04CF | 0.31 | 0.13 | - | 2.34 | 2.34 | * |
| T4F21.15 | 2B-156D | 2.98 | 1C-04CF | 0.67 | 0.67 | - | 2.62 | 2.62 | * |
| T4F21.17 | 2B-156D | 1.66 | 1C-04CF | 1.04 | 0.00 |  | 2.41 | 2.41 | * |
| T4F21.18 | 2B-156D | 1.66 | 1C-04CF | 1.03 | 0.64 | - | 2.71 | 2.71 | * |
| T4F21.19 | 2B-156D | 1.77 | 1C-04CF | 2.08 | 2.08 | + | 4.57 | 4.57 | * |
| T4F21.2 | 2B-156D | 2.00 | 1C-04CF | 1.77 | 1.77 | + | 3.83 | 3.83 | * |
| T4F22.1 | 29-EE70 | 1.89 | 1C-04CF | 1.47 | 0.00 |  | 2.57 | 2.57 | * |
| T4F22.2 | 29-EE70 | 2.47 | 1C-04CF | 1.68 | 0.13 | - | 3.05 | 3.05 | * |
| T4F22.3 | 29-EE70 | 3.66 | 1C-04CF | 1.09 | 1.09 | + | 3.20 | 3.20 | * |
| T4F22.4 | 29-EE70 | 3.86 | 1C-04CF | 0.36 | 0.00 |  | 2.26 | 2.26 | * |
| T4F22.5 | 29-EE70 | 4.17 | 1C-04CF | 2.26 | 0.00 |  | 3.64 | 3.64 | * |
| T4F22.6 | 29-EE70 | 3.06 | 1C-04CF | 0.15 | 0.00 |  | 1.78 | 1.78 | + |
| T4F22.7 | 29-EE70 | 5.40 | 1C-04CF | 1.53 | 1.53 | + | 3.50 | 3.50 | * |
| T4F22.8 | 29-EE70 | 5.26 | 1C-04CF | 1.09 | 1.09 | + | 3.21 | 3.21 | * |
| T4F25.1 | 2A-01E6 | 5.70 | 24-8506 | 4.20 | 4.20 | * | 5.81 | 5.81 | * |
| T4F25.2 | 2A-01E6 | 5.70 | 1C-04CF | 0.62 | 0.62 | - | 3.36 | 3.36 | * |
| T4F25.3 | 2A-01E6 | 5.00 | 1C-04CF | 0.45 | 0.45 | - | 3.20 | 3.20 | * |
| T4F25.4 | 2A-01E6 | 5.00 | 1C-04CF | 0.45 | 0.45 | - | 3.20 | 3.20 | * |
| T4F25.6 | 2A-01E6 | 5.00 | 1C-04CF | 2.30 | 2.30 | + | 5.07 | 5.07 | * |
| T4F25.7 | 2A-01E6 | 5.75 | 24-8506 | 2.10 | 2.10 | + | 4.17 | 4.17 | * |
| T4F25.8 | 2A-01E6 | 5.00 | 1C-04CF | -0.02 | 0.00 |  | 2.72 | 2.72 | * |
| T4F25.9 | 2A-01E6 | 5.00 | 1C-04CF | -0.16 | 0.00 |  | 2.58 | 2.58 | * |
| T5F1.1 | 2B-156D | 5.30 | 24-8506 | 3.09 | 3.09 | * | 5.18 | 5.18 | * |
| T5F1.2 | 2B-156D | 4.41 | 24-8506 | 0.80 | 0.00 |  | 3.02 | 3.02 | * |
| T5F1.3 | 2B-156D | 0.95 | 1C-04CF | 2.85 | 0.50 | + | 4.66 | 4.66 | * |
| T5F1.4 | 2B-156D | 1.97 | 1C-04CF | 1.52 | 1.52 | + | 3.32 | 3.32 | * |
| T5F1.5 | 2B-156D | 2.82 | 24-8506 | 3.58 | 3.58 | * | 5.85 | 5.85 | * |
| T5F1.6 | 2B-156D | 1.50 | 24-8506 | 3.63 | 1.96 | + | 5.77 | 5.77 | * |
| T5F1.7 | 2B-156D | 5.30 | 1C-04CF | 1.23 | 1.23 | + | 3.29 | 3.29 | * |
| T5F10.2 | 29-EF52 | 7.89 | 1C-04CF | 1.28 | 1.28 | + | 3.34 | 3.34 | * |
| T5F10.3 | 29-EF52 | 6.52 | 1C-04CF | 1.52 | 0.86 | + | 4.27 | 4.27 | * |
| T5F10.4 | 29-EF52 | 5.12 | 1C-04CF | 3.00 | 3.00 | * | 4.28 | 4.28 | * |
| T5F10.6 | 29-EF52 | 4.10 | 1C-04CF | 2.38 | 2.38 | + | 3.65 | 3.65 | * |
| T5F2.1 | 1C-08B1 | 3.15 | 1C-04CF | 1.52 | 0.00 |  | 2.90 | 2.90 | * |
| T5F2.2 | 1C-08B1 | 3.26 | 24-8F52 | 2.35 | 0.19 | - | 3.39 | 1.38 | * |
| T5F2.3 | 1C-08B1 | 3.33 | 1C-04CF | 0.84 | 0.00 |  | 2.36 | 2.36 | * |
| T5F2.4 | 1C-08B1 | 2.99 | 1C-04CF | 1.32 | 1.32 | + | 2.53 | 2.53 | * |
| T5F2.5 | 1C-08B1 | 3.62 | 1C-04CF | 1.91 | 0.00 |  | 2.86 | 2.86 | * |
| T5F2.6 | 1C-08B1 | 2.99 | 1C-04CF | 1.32 | 1.32 | + | 2.53 | 2.53 | * |
| T5F2.7 | 1C-08B1 | 1.55 | 1C-04CF | 2.48 | 2.48 | + | 4.66 | 4.66 | * |
| T5F3.1 | 24-8AA5 | 2.16 | 24-8506 | 4.14 | 4.14 | * | 5.17 | 5.17 | * |
| T5F3.2 | 24-8AA5 | 1.23 | 24-8506 | 4.20 | 2.29 | + | 6.77 | 6.77 | * |
| T5F3.3 | 24-8AA5 | 2.53 | 24-8506 | 4.45 | 4.45 | * | 6.16 | 6.16 | * |
| T5F3.4 | 24-8AA5 | 2.56 | 31-B7F0 | 2.17 | 0.00 |  | 3.51 | 0.00 | + |
| T5F3.5 | 24-8AA5 | 2.82 | 24-8506 | 1.80 | 0.00 |  | 4.32 | 4.32 | * |
| T5F3.6 | 24-8AA5 | 2.89 | 24-8506 | 3.10 | 3.10 | * | 4.51 | 4.51 | * |
| T5F3.8 | 24-8AA5 | 3.47 | 24-8506 | 2.04 | 0.13 | - | 3.91 | 3.91 | * |
| T5F4.10 | 29-FA22 | 3.59 | 1C-04CF | 2.20 | 0.00 |  | 4.27 | 4.27 | * |
| T5F4.11 | 29-FA22 | 1.93 | 1C-04CF | 3.70 | 3.70 | * | 5.50 | 5.50 | * |
| T5F4.2 | 29-FA22 | 0.63 | 1C-04CF | 2.66 | 0.47 | + | 4.61 | 4.61 | * |
| T5F4.3 | 29-FA22 | 4.21 | 1C-04CF | 3.43 | 3.43 | * | 5.51 | 5.51 | * |
| T5F4.5 | 29-FA22 | 2.01 | 1C-04CF | 1.68 | 1.68 | + | 3.63 | 3.63 | * |
| T5F4.6 | 29-FA22 | 4.21 | 1C-04CF | 2.46 | 2.46 | + | 4.53 | 4.53 | * |
| T5F4.7 | 29-FA22 | 3.01 | 1C-04CF | 0.39 | 0.39 | + | 2.44 | 2.44 | * |
| T5F4.8 | 29-FA22 | 2.33 | 1C-04CF | 2.39 | 2.39 | + | 4.88 | 4.88 | * |
| T5F4.9 | 29-FA22 | 2.78 | 1C-04CF | 1.01 | 1.01 | + | 2.53 | 2.53 | * |
| T5F5.2 | 29-EE70 | 3.72 | 1C-04CF | 1.87 | 1.07 | + | 4.56 | 4.56 | * |
| T5F5.3 | 29-EE70 | 2.38 | 1C-04CF | 1.58 | 1.58 | + | 3.64 | 3.64 | * |
| T5F5.5 | 29-EE70 | 3.35 | 1C-04CF | 1.47 | 1.47 | + | 3.69 | 3.69 | * |
| T5F6.1 | 1B-E62F | 0.39 | 1B-F658 | 4.51 | 4.51 | * | 6.22 | 6.22 | * |
| T5F6.10 | 1B-E62F | 2.76 | 1B-F658 | 3.35 | 3.35 | * | 5.45 | 5.45 | * |
| T5F6.2 | 1B-E62F | 4.36 | 1B-F658 | 2.52 | 2.52 | + | 3.90 | 3.90 | * |
| T5F6.3 | 1B-E62F | 3.98 | 1B-F658 | 0.99 | 0.99 | + | 2.06 | 2.06 | * |
| T5F6.6 | 1B-E62F | 2.69 | 1B-F658 | 1.96 | 1.96 | + | 2.87 | 2.87 | * |
| T5F6.7 | 1B-E62F | 2.36 | 1B-F658 | 2.52 | 2.52 | + | 3.89 | 3.89 | * |
| T5F6.8 | 1B-E62F | 1.76 | 1B-F658 | 3.03 | 3.03 | * | 3.94 | 3.94 | * |
| T5F6.9 | 1B-E62F | 1.07 | 1B-F658 | 3.15 | 3.15 | * | 4.36 | 4.36 | * |
| T5F7.2 | 2A-0359 | 6.34 | 1B-F658 | 3.51 | 3.20 | * | 5.40 | 5.40 | * |
| T5F7.4 | 2A-0359 | 6.12 | 1B-F658 | 1.23 | 0.43 | + | 3.11 | 3.11 | * |
| T5F7.5 | 2A-0359 | 7.44 | 1B-F658 | 1.12 | 1.12 | + | 3.87 | 3.87 | * |
| T5F7.6 | 2A-0359 | 6.34 | 1B-F658 | 1.73 | 1.43 | + | 3.62 | 3.62 | * |
| T5F7.7 | 2A-0359 | 7.68 | 1B-F658 | 4.16 | 4.16 | * | 6.25 | 6.25 | * |
| T5F8.1 | 34-E03B | 4.46 | 1B-F658 | 1.12 | 0.00 |  | 3.86 | 3.86 | * |
| T5F8.2 | 34-E03B | 4.72 | 1B-F658 | 1.62 | 1.62 | + | 4.11 | 4.11 | * |
| T5F8.3 | 34-E03B | 5.44 | 1B-F658 | 3.06 | 3.06 | * | 4.76 | 4.76 | * |
| T5F8.4 | 34-E03B | 6.21 | 1B-F658 | 2.71 | 2.71 | * | 4.84 | 4.84 | * |
| T5F8.5 | 34-E03B | 5.06 | 1B-F658 | 2.31 | 2.31 | + | 4.38 | 4.38 | * |
| T5F8.6 | 34-E03B | 3.70 | 1B-F658 | 3.35 | 3.35 | * | 5.73 | 5.73 | * |
| T5F8.7 | 34-E03B | 4.39 | 1B-F658 | 2.90 | 2.90 | * | 4.97 | 4.97 | * |
| T5F8.8 | 34-E03B | 4.30 | 1B-F658 | 2.35 | 2.35 | + | 3.45 | 3.45 | * |

Table S6. Detailed summary table of mating success with all males included. Within trials males are ordered by decreasing mating success (“MS”).

| trial | male ID | SL (cm) | Weight (g) | Condition | tag reader number | # offspring | # females | total # offispring | Male MS |
| --- | --- | --- | --- | --- | --- | --- | --- | --- | --- |
|  |  |  |  | factor |  |  |  |  |  |
| 1 | 1B-E516 | NA | 27.20 | NA | 1 | 26 | 3 | 56 | 0.46 |
|  | 24-82B0 | NA | 27.35 | NA | 2 | 13 | 3 |  | 0.23 |
|  | 24-987B | NA | 30.61 | NA | 3 | 12 | 3 |  | 0.21 |
|  | 24-90DA | NA | 31.26 | NA | 4B | 5 | 2 |  | 0.09 |
|  | 1A-2DBF | NA | 28.54 | NA | no bower | 0 | 0 |  | 0.00 |
|  | 1B-E404 | NA | 35.16 | NA | no bower | 0 | 0 |  | 0.00 |
|  | 1B-E918 | NA | 25.90 | NA | 4A | 0 | 0 |  | 0.00 |
|  | 24-7FDA | NA | 19.67 | NA | no bower | 0 | 0 |  | 0.00 |
|  | 24-8799 | NA | 36.05 | NA | no bower | 0 | 0 |  | 0.00 |
|  | 24-8A4E | NA | 23.43 | NA | no bower | 0 | 0 |  | 0.00 |
|  | 24-90E2 | NA | 20.50 | NA | no bower | 0 | 0 |  | 0.00 |
|  | 2A-01A7 | NA | 21.88 | NA | no bower | 0 | 0 |  | 0.00 |
|  | 2A-F8B7 | NA | 22.70 | NA | no bower | 0 | 0 |  | 0.00 |
|  | 34-BCEC | NA | 19.50 | NA | no bower | 0 | 0 |  | 0.00 |
|  | 38-13F6 | NA | 42.23 | NA | no bower | 0 | 0 |  | 0.00 |
| 2 | 24-8799 | NA | 36.05 | NA | 4A | 25 | 4 | 38 | 0.66 |
|  | 24-987B | NA | 30.61 | NA | 1, 2A, 4B | 8 | 1 |  | 0.21 |
|  | 24-82B0 | NA | 27.35 | NA | 3 | 3 | 1 |  | 0.08 |
|  | 1B-E918 | NA | 25.90 | NA | no bower | 1 | 1 |  | 0.03 |
|  | 34-BCEC | NA | 19.50 | NA | 1 | 1 | 1 |  | 0.03 |
|  | 1B-E404 | NA | 35.16 | NA | no bower | 0 | 0 |  | 0.00 |
|  | 1B-E516 | NA | 27.20 | NA | no bower | 0 | 0 |  | 0.00 |
|  | 24-7FDA | NA | 19.67 | NA | 3 | 0 | 0 |  | 0.00 |
|  | 24-8A4E | NA | 23.43 | NA | no bower | 0 | 0 |  | 0.00 |
|  | 24-90E2 | NA | 20.50 | NA | no bower | 0 | 0 |  | 0.00 |
|  | 2A-01A7 | NA | 21.88 | NA | 1, 5 | 0 | 0 |  | 0.00 |
|  | 2A-F8B7 | NA | 22.70 | NA | no bower | 0 | 0 |  | 0.00 |
|  | 38-13F6 | NA | 42.23 | NA | 2B | 0 | 0 |  | 0.00 |
| 3 | 24-8799 | 10.50 | 31.75 | 4.82 | 1 | 20 | 5 | 45 | 0.44 |
|  | 24-7FDA | 10.40 | 31.28 | 4.88 | 3 | 19 | 4 |  | 0.42 |
|  | 24-987B | 9.80 | 24.10 | 4.43 | no bower | 3 | 1 |  | 0.07 |
|  | 2A-01A7 | 10.00 | 28.88 | 5.02 | 2 | 2 | 2 |  | 0.04 |
|  | 29-F20E | 9.50 | 27.67 | 5.54 | no bower | 1 | 1 |  | 0.02 |
|  | 1B-E404 | 11.20 | 38.57 | 4.90 | no bower | 0 | 0 |  | 0.00 |
|  | 1B-E516 | 10.50 | 33.38 | 5.07 | no bower | 0 | 0 |  | 0.00 |
|  | 1B-E918 | 10.70 | 27.24 | 3.93 | no bower | 0 | 0 |  | 0.00 |
|  | 24-82B0 | 10.30 | 27.65 | 4.43 | no bower | 0 | 0 |  | 0.00 |
|  | 24-8601 | 9.40 | 21.86 | 4.51 | no bower | 0 | 0 |  | 0.00 |
|  | 24-8A4E | 10.40 | 32.69 | 5.10 | no bower | 0 | 0 |  | 0.00 |
|  | 24-90E2 | 10.20 | 26.06 | 4.29 | no bower | 0 | 0 |  | 0.00 |
|  | 2A-F8B7 | 9.90 | 31.61 | 5.65 | 2 | 0 | 0 |  | 0.00 |
|  | 34-BCEC | 9.80 | 24.56 | 4.51 | 2 | 0 | 0 |  | 0.00 |
|  | 38-13F6 | 11.50 | 38.88 | 4.59 | no bower | 0 | 0 |  | 0.00 |
| 4 | 1C-04CF | 9.80 | 23.37 | 4.29 | 2 | 35 | 6 | 42 | 0.83 |
|  | 24-8506 | 10.00 | 25.79 | 4.48 | 1, 3 | 6 | 2 |  | 0.14 |
|  | 24-9889 | 8.70 | 20.39 | 5.20 | no bower | 1 | 1 |  | 0.02 |
|  | 1A-3CC2 | 9.00 | 19.88 | 4.62 | no bower | 0 | 0 |  | 0.00 |
|  | 1B-E4BA | 9.70 | 20.95 | 3.96 | no bower | 0 | 0 |  | 0.00 |
|  | 1B-F658 | 9.30 | 21.02 | 4.46 | no bower | 0 | 0 |  | 0.00 |
|  | 1C-A29A | 10.30 | 28.33 | 4.54 | no bower | 0 | 0 |  | 0.00 |
|  | 24-78D0 | 8.50 | 16.26 | 4.43 | no bower | 0 | 0 |  | 0.00 |
|  | 29-F84E | 8.10 | 15.91 | 4.95 | no bower | 0 | 0 |  | 0.00 |
|  | 2A-0855 | 9.70 | 23.37 | 4.42 | no bower | 0 | 0 |  | 0.00 |
|  | 2A-F497 | 10.30 | 24.39 | 3.91 | no bower | 0 | 0 |  | 0.00 |
|  | 2B-11FC | 9.50 | 23.03 | 4.61 | no bower | 0 | 0 |  | 0.00 |
|  | 31-B7F0 | 9.30 | 21.98 | 4.67 | no bower | 0 | 0 |  | 0.00 |
|  | 24-9579 | 10.20 | 34.51 | 5.68 | 1 | 0 | 0 |  | 0.00 |
|  | 38-0E9B | 9.10 | 21.53 | 4.85 | no bower | 0 | 0 |  | 0.00 |
| 5 | 1C-04CF | 10.40 | 31.34 | 4.89 | 3 | 25 | 5 | 58 | 0.43 |
|  | 1B-F658 | 10.60 | 38.09 | 5.64 | 3 | 21 | 3 |  | 0.36 |
|  | 24-8506 | 11.00 | 37.21 | 4.97 | 1 | 10 | 2 |  | 0.17 |
|  | 24-8F52 | 9.40 | 25.52 | 5.26 | no bower | 1 | 1 |  | 0.02 |
|  | 31-B7F0 | 10.40 | 30.50 | 4.76 | no bower | 1 | 1 |  | 0.02 |
|  | 1A-3CC2 | 10.40 | 31.81 | 4.96 | no bower | 0 | 0 |  | 0.00 |
|  | 1B-F6A9 | 9.00 | 23.45 | 5.45 | no bower | 0 | 0 |  | 0.00 |
|  | 1C-A29A | 10.60 | 30.34 | 4.49 | 2 | 0 | 0 |  | 0.00 |
|  | 24-78D0 | 8.70 | 17.12 | 4.37 | no bower | 0 | 0 |  | 0.00 |
|  | 29-F84E | 9.30 | 22.73 | 4.83 | no bower | 0 | 0 |  | 0.00 |
|  | 2A-0855 | 10.90 | 36.30 | 4.97 | no bower | 0 | 0 |  | 0.00 |
|  | 2A-0C48 | 8.30 | 17.59 | 5.11 | no bower | 0 | 0 |  | 0.00 |
|  | 2B-0916 | 9.90 | 27.07 | 4.84 | no bower | 0 | 0 |  | 0.00 |
|  | 2B-11FC | 10.30 | 31.19 | 5.00 | 2 | 0 | 0 |  | 0.00 |
|  | 38-0E9B | 9.90 | 26.67 | 4.77 | no bower | 0 | 0 |  | 0.00 |

Video S1 – Overview of the pool used for the mesocosm experiment and zoom-in of male *Nyassachromis* cf. *microcephalus* defending and building his bower.
